# Clinical and Prognostic Implications of Transcription Factor SOX4 in Patients with Colon Cancer

**DOI:** 10.1371/journal.pone.0067128

**Published:** 2013-06-27

**Authors:** Chun-Mao Lin, Chia-Lang Fang, You-Cheng Hseu, Chun-Liang Chen, Jin-Wun Wang, Sheng-Lung Hsu, Ming-Dao Tu, Shih-Ting Hung, Chein Tai, Yih-Huei Uen, Kai-Yuan Lin

**Affiliations:** 1 Department of Biochemistry, School of Medicine, College of Medicine, Taipei Medical University, Taipei, Taiwan; 2 Orthopedics Research Center, Taipei Medical University Hospital, Taipei, Taiwan; 3 Department of Pathology, School of Medicine, College of Medicine, Taipei Medical University, Taipei, Taiwan; 4 Department of Pathology, Wan Fang Hospital, Taipei Medical University, Taipei, Taiwan; 5 Department of Cosmeceutics, China Medical University, Taichung, Taiwan; 6 Department of Molecular and Cellular Oncology, The University of Texas MD Anderson Cancer Center, Houston, Texas, United States of America; 7 Department of Surgery, Chi Mei Hospital Chiali, Tainan, Taiwan; 8 Department of Family Medicine, Chi Mei Hospital Chiali, Tainan, Taiwan; 9 Department of Medical Research, Chi Mei Medical Center, Tainan, Taiwan; 10 Department of Biotechnology, Southern Taiwan University of Science and Technology, Tainan, Taiwan; 11 Superintendent’s Office, Chi Mei Hospital Chiali, Tainan, Taiwan; 12 Institute of Biomedical Engineering, Southern Taiwan University of Science and Technology, Tainan, Taiwan; 13 Department of Biotechnology, Chia Nan University of Pharmacy and Science, Tainan, Taiwan; Enzo Life Sciences, Inc., United States of America

## Abstract

Colon cancer is one of the most common malignant cancers worldwide but the current therapeutic approaches for advanced colon cancer are less efficient. This study investigated associations between the expression of nuclear transcription factor SOX4 and various clinicopathologic parameters as well as patients’ survival. Expression levels of nuclear SOX4 were analyzed by immunohistochemistry; the data comprised colon tissues from 263 patients with colon cancer. Paired *t* tests were used to analyze the differences in nuclear SOX4 expression between tumor and non-tumor tissues from each patient. Two-tailed Χ^2^ tests were performed to determine whether the differences in nuclear SOX4 expression and clinicopathologic parameters were significant. Time-to-event endpoints for clinicopathologic parameters were plotted using the Kaplan-Meier method, and statistical significance was determined using univariate log-rank tests. Cox proportional hazard model was used for multivariate analysis to determine the independence of prognostic effects of nuclear SOX4 expression. Overexpression of nuclear SOX4 was significantly correlated with depth of invasion (*P* = 0.0041), distant metastasis (*P*<0.0001), and stage (*P* = 0.0001). Patients who displayed high expression levels of nuclear SOX4 achieved a significantly poorer disease-free survival rate, compared with patients with low SOX4 expression levels (*P*<0.001). Univariate Cox regression analysis showed that overexpression of nuclear SOX4 was a clear prognostic marker for colon cancer (*P* = 0.001). Overexpression of nuclear SOX4 may be used as a marker to predict the outcome of patients with colon cancer.

## Introduction

Colon cancer is one of the most frequent cancers and a common cause of cancer-related deaths [1]. In Taiwan, colon cancer ranks as the most frequently diagnosed malignancy and causes more than 4900 deaths annually (http://www.doh.gov.tw/statistic/index.htm; accessed in September 2012). In spite of the current surgical techniques and chemotherapy that have made significant improvements, due to the poor prognosis and distant invasion and migration, the overall incidence of colon cancer is approximately 5% and the 5-year survival rate of colon cancer patients is very low [2]. Thus, the identification of new targets for the development of non-conventional treatments is urgent and will take advantage of progress in the broad and deep understanding of the molecular pathogenesis of colon cancer.

Among the prognostic markers now available for colon cancer, the most important is the American Joint Committee on Cancer (AJCC) stage determined by the depth of invasion, the involvement of the lymph nodes, and distant metastasis. However, the prognosis varies even among patients at the same stage. In recent decades, several studies have suggested that genetic alterations may play a role in the development and progression of colon cancer [3], [4]. Studies in molecular pathology may help in understanding the disease pathogenesis and might also reveal useful prognostic molecular markers. Some suggested biological prognostic markers include overexpression of protein kinase CK2, vascular endothelial growth factor (VEGF), enhancer of zeste homologue 2 and transglutaminase 2 [5]–[8].

In humans, the sex-determining region Y (SRY) box family, also referred to as the SOX family, comprises 20 highly conserved transcription factors that play important roles in development. These transcription factors are defined by a conserved signature sequence in the high-mobility group (HMG) DNA-binding domain (DBD) [9], [10]. SOX4 is a 47-kDa protein that is encoded by a single exon gene, which is highly conserved in vertebrates. In mice, SOX4 is specifically expressed in the ovary, testis, mammary gland, and thymus and in mouse T and pre-B lymphocytic cell lines [11]. In addition, SOX4 is essential for the development of the heart, lymphocytes, and thymocytes, and SOX4-null mice die from cardiac defects [12]. The proliferative capacity of B-cell progenitors is severely decreased in cells from SOX4 knockout mice [13].

The clinical importance of SOX4 has gained increasing attention in recent years, with numerous reports suggesting that SOX4 may contribute to tumor progression. Three independent studies screening for important oncogenes showed that SOX4 is frequently altered through retroviral insertions [14]–[16]. The murine leukemia virus typically targeted SOX4, and stabilized the SOX4 message to produce B-cell lymphomas that displayed increased SOX4 message levels [16]. Increased SOX4 expression is associated with tumors of the bladder, prostate, and with non-small-cell lung tumors [17]–[19]. However, the role of SOX4 in such tumors is not fully understood and the reported data have shown certain contradictions. Whereas SOX4 knockdown resulted in apoptosis of ACC3 adenoid cystic carcinoma cells, SOX4 overexpression promoted cell cycle arrest and apoptosis of HCT116 colon carcinoma cells [20], [21]. The microRNA (miRNA) miR-335 inhibited metastatic cell invasion and acted, at least in part, through targeting *sox4* and its putative target *TNC*, which encodes an extracellular matrix component implicated in cell migration [22]. By contrast, the higher the level of SOX4 expression, the better the prognosis for patients with medulloblastomas and other tumor types [23]. Thus, SOX4 might exert different effects on tumor cells depending on the context and primary transformation mechanism; further studies are warranted to clarify this issue.

To date, the prognostic significance of nuclear SOX4 expression levels in human colon cancer has not been established. This study investigated the associations between nuclear SOX4 expression and clinicopathologic parameters, and evaluated the significance of nuclear SOX4 in predicting the prognosis for patients with colon cancer.

## Materials and Methods

### Ethics Statement

The institutional review board at Chi Mei Medical Center approved the tissue acquisition protocol for the immunohistochemical and immunoblotting study. Written informed consent was obtained from each participant before tissue acquisition.

### Participants and Specimens

We collected 263 consecutive colon cancer cases from the medical files of Chi Mei Medical Center in Taiwan. All patients included in our study group were treated between 1998 and 2005, and had received colectomy with lymph node dissection. All cases got tissue proof of adenocarcinoma via colonoscopic biopsy before operation. Completeness of surgical resection was achieved in all cases, and pathological examination revealed no tumor involvement of the resection margins in surgical specimens. None of our study patients had received preoperative chemotherapy and/or radiotherapy. The non-tumor portion was obtained from grossly normal colon mucosa, separate from the tumor, in resected colon specimen. Clinicopathologic parameters of colon cancer were determined according to the AJCC classification. The follow-up duration for disease-free survival was defined as the period between the operation date and the day of relapse, according to the patient’s chart. For each patient, we analyzed a pair of tumor and non-tumor colon tissues to determine the nuclear SOX4 expression.

### Immunohistochemical Analysis

Nuclear SOX4 expression was analyzed by immunohistochemistry. Paraffin-embedded tissue blocks were sectioned at 5 µm and transferred to microscope slides (Muto Pure Chemicals Co. Ltd., Tokyo, Japan). Breast tissue was used as a positive control for SOX4. The negative control entailed omission of the primary antibody and incubation with phosphate buffer saline. Sections were dewaxed with xylene, followed by rehydration in graded alcohols. Deparaffinized sections were incubated in pH 6.0 citrate buffer for 40 min at 95°C on a hotplate to retrieve antigens. Further antigen blocking was performed using Dako REAL Peroxidase-Blocking Solution (Dako North America Inc., Carpinteria, CA) for 5 min. The slides were subsequently incubated with primary antibody: polyclonal anti-SOX4 (Life-Span, Victoria, Canada) for 1 hour at room temperature, at a dilution of 1∶3200. Detection of the immunoreactive staining was conducted using the avidin-biotin-peroxidase complex method according to the manufacturer’s instructions. A sensitive Dako REAL EnVision Detection System (Dako North America Inc., Carpinteria, CA) was used. After incubation with diaminobenzidine for 5 minutes, the sections were counterstained with hematoxylin and mounted in Dako Faramount Aqueous Mounting Medium (Dako North America Inc., Carpinteria, CA) for microscopic interpretation. Immunoreactivity was semiquantitatively scored based on intensity of immunostaining: 0, no staining; 1, weak staining; 2, moderate staining; 3, strong staining. Sections with a score of 0 or 1 displayed low expression of SOX4, whereas those that scored 2 or 3 were defined as having high expression or overexpression of SOX4. Clinical data collection and immunohistochemical analysis were performed independently of each other, in an investigator-blinded study.

### Cell Culture

Human normal (FHC) and colon cancer cell lines (DLD-1 and WiDr) were obtained from the American Type Culture Collection (ATCC; Manassas, VA, USA). Cell lines were authenticated by the ATCC cell biology program, and were passaged for no longer than 6 months before new cells were brought out of the frozen state or a new cell aliquot was purchased from ATCC. Cells were cultured in DMEM/F-12 (FHC), RPMI-1640 (DLD-1), or MEM (WiDr) media supplemented with 10% fetal bovine serum, 100 units/mL penicillin G, 100 µg/mL streptomycin sulfate, and 250 ng/mL amphotericin B.

### Nuclear Protein Preparation

Nuclear proteins were extracted using NE-PER Nuclear Extraction Reagent (Pierce Biotechnology, Rockford, IL), according to the manufacturer’s instructions. The samples were stored at −80°C until used. The protein concentration was determined using a BCA Protein Assay Kit (Pierce Biotechnology) with bovine serum albumin as a standard.

### Immunoblotting

Denatured protein samples were subjected to 12% SDS-PAGE. Proteins were transferred to nitrocellulose membranes, and blocked blots were incubated at 4°C overnight with anti-SOX4 polyclonal antibody (1∶1000 dilution). TATA binding protein was used as an internal control for equal protein loading. Blots were further incubated with secondary antibodies conjugated with peroxidase (Sigma, St. Louis, MO) for 1 h at room temperature. They were then incubated with SuperSignal West Femto Maximun Sensitivity Substrate (Pierce Biotechnology, Inc., Rockford, IL), and exposed to a Fuji medical x-ray film (Fuji Photo Film Co., Tokyo, Japan). Image processing was performed using Fuji Image Gauge software.

### Statistical Analysis

Paired *t* tests were used to assess the difference in nuclear SOX4 expression between tumor and non-tumor tissues for each patient. We examined several clinicopathologic parameters: age, gender, depth of invasion, nodal status, distant metastasis, stage, degree of differentiation, vascular permeation and perineural invasion. The association between nuclear SOX4 expression and each clinicopathologic parameter was examined using χ^2^ test. The time-to-event endpoints for all clinicopathologic parameters were plotted by the Kaplan-Meier method, and the degree of significance was calculated by the univariate log-rank test. Parameters that emerged as significant (*P*≤0.05) in univariate analysis were entered as variables in the multivariate Cox regression model, and hazard ratio (HR) and independence of prognostic impact could be determined in a stepwise backward fashion. All data were analyzed using SPSS software version 17.0 (SPSS, Chicago, IL). A *P* value of <0.05 was considered significant.

## Results

### Demographics

This study enrolled 263 patients with colon cancer, 154 of whom were men and 109 were women (Table 1). The patients’ ages ranged from 21 to 92 years at first diagnosis (mean ± standarad deviation (S.D.): 68.0±12.9 years). Based on the AJCC classification, 25 patients were at stage I, 91 were at stage II, 108 were at stage III, and 39 were at stage IV. The follow-up period for all patients ranged from 0 to 146 months (mean ± S.D.: 58.6±40.2 months). During follow-up, 116 patients died of colon cancer.

**Table 1:** Demographic data and survival in different stages of colon cancer according to the AJCC classification.

	Stage I	Stage II	Stage III	Stage IV	Total
	(n = 25)	(n = 91)	(n = 108)	(n = 39)	(n = 263)
Gender					
Male	15	57	59	23	154
Female	10	34	49	16	109
Age (years)*	66.9 (10.3)	69.1 (12.7)	68.4 (12.6)	65.3 (15.9)	68.0 (12.9)
Follow-up period	77.6 (32.5)	68.0 (38.8)	61.9 (38.5)	15.5 (18.3)	58.6 (40.2)
(months)*					
Survival					
Yes	20	61	64	2	147
No	5	30	44	37	116

* Age and follow-up period are mean (S.D.).

### Nuclear SOX4 Expression was Upregulated and Associated with Clinicopathologic Parameters in Colon Cancer

**Figure 1:**
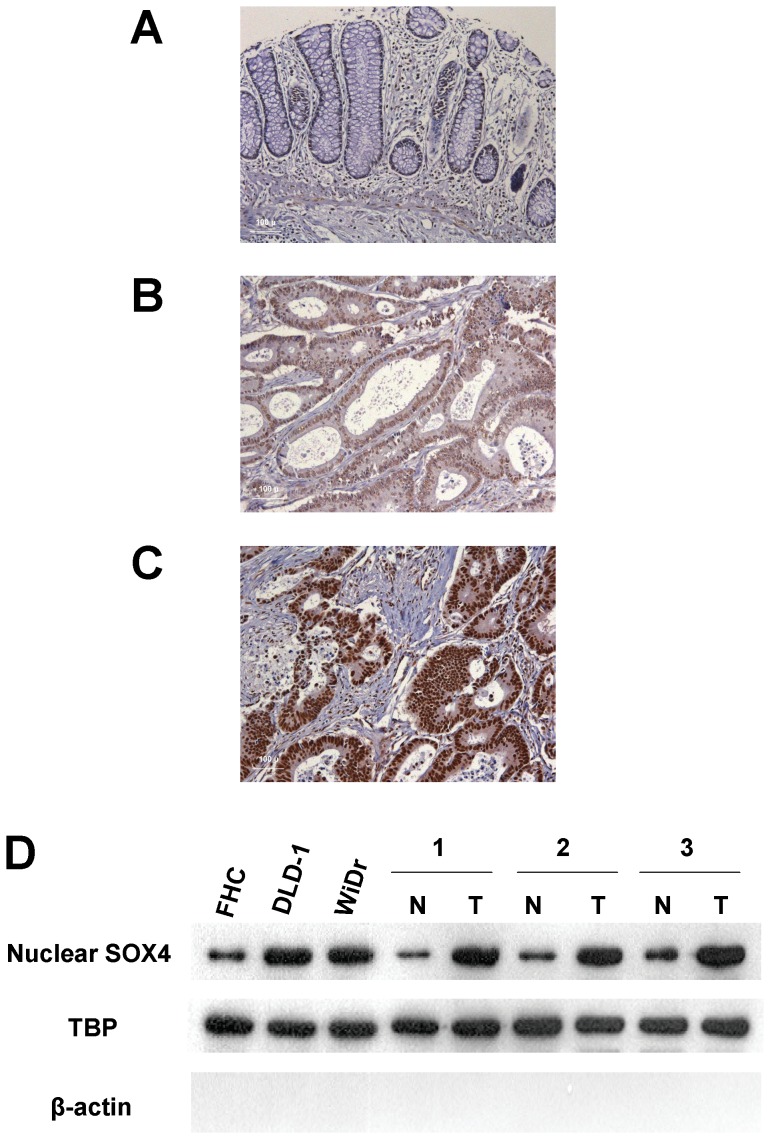
Expression of SOX4 in colon tissues and cell lines. Panels A to C: Colon cancer specimens analyzed by immunohistochemistry with an antibody against SOX4. Panel A shows a sample without SOX4 expression; Panel B shows a sample with low expression level of SOX4; Panel C shows a sample with high expression level of SOX4. Panel D: Nuclear SOX4 protein expression was examined in 3 colon cells and 3 non-tumor/tumor pairs of colon tissues. Magnification: 200×.

We used immunohistochemical analysis to investigate the expression of nuclear SOX4 in tissues obtained from our study patients (Figures 1A to C). Nuclear SOX4 expression was significantly higher in tumor tissues than in non-tumor tissues (*P*<0.001). Overexpression of nuclear SOX4 (scores of 2 or 3) was observed in 119 of the 263 patients (45.2%). Western blot analysis also demonstrated that the expression of SOX4 was substantially increased in colon cancer cells and tissues when compared with normal cells and tissues (Figure 1D). Additionally, quantitative real-time PCR analysis demonstrated that the expression of SOX4 mRNA was substantially increased in tumor tissues when compared with non-tumor tissues (Table 2). As shown in Table 3, overexpression of nuclear SOX4 correlated significantly with the following parameters: depth of invasion (*P* = 0.0041), distant metastasis (*P*<0.0001), and stage (*P* = 0.0001). No significant association emerged between overexpression of nuclear SOX4 and age, gender, nodal status, degree of differentiation, vascular invasion, or perineural invasion.

**Table 2:** Quantification of SOX4 mRNA expression by quantitative real-time PCR in 10 tumor and non-tumor pairs of colon tissues.

	Non-tumor			Tumor		
No.	SOX4	β-actin	Δ*C_non-tumor_*	SOX4	β-actin	Δ*C_tumor_*
S0063	28.56	20.15	8.41	25.12	19.58	5.54
S0423	29.14	20.18	8.96	26.54	20.44	6.10
S0475	31.27	23.67	7.60	28.49	23.12	5.37
S0480	28.33	19.50	8.83	25.48	19.87	5.61
S0485	29.51	20.77	8.74	26.97	21.03	5.94
S0597	32.04	24.18	7.86	30.26	23.89	6.37
S0641	31.25	22.54	8.71	29.07	22.81	6.26
S0680	27.63	18.69	8.94	24.23	18.25	5.98
S0705	31.96	23.22	8.74	30.66	24.35	6.31
S0708	28.53	20.37	8.16	26.59	19.94	6.65

**Table 3:** Nuclear SOX4 expression in colon cancer and its association with clinicopathologic parameters.

		Nuclear SOX4 expression		
		Score = 0 or 1	Score = 2 or 3	
		(n = 144)	(n = 119)	
Variable	n	<1.54	≥1.54	*P* *
Age (yr)				0.2313
≥68	162	84	78	
<68	101	60	41	
Gender				0.2774
Male	154	80	74	
Female	109	64	45	
Depth of invasion				0.0041
T1	11	9	2	
T2	22	16	6	
T3	198	109	89	
T4	32	10	22	
Nodal status				0.3198
N0	126	73	53	
N1+ N2+ N3	137	71	66	
Distant metastasis				<0.0001
Absent	224	134	90	
Present	39	10	29	
Stage				0.0001
I	25	20	5	
II	91	51	40	
III	108	63	45	
IV	39	10	29	
Degree of differentiation				0.0513
Poor	16	5	11	
Well to moderate	247	139	108	
Vascular invasion				0.2050
Absent	124	73	51	
Present	139	71	68	
Perineural invasion				0.7162
Absent	206	114	92	
Present	57	30	27	

* All statistical tests were two-tailed and the significance level was *P*<0.05.

### Overexpression of Nuclear SOX4 as a Prognostic Marker for Colon Cancer

**Figure 2:**
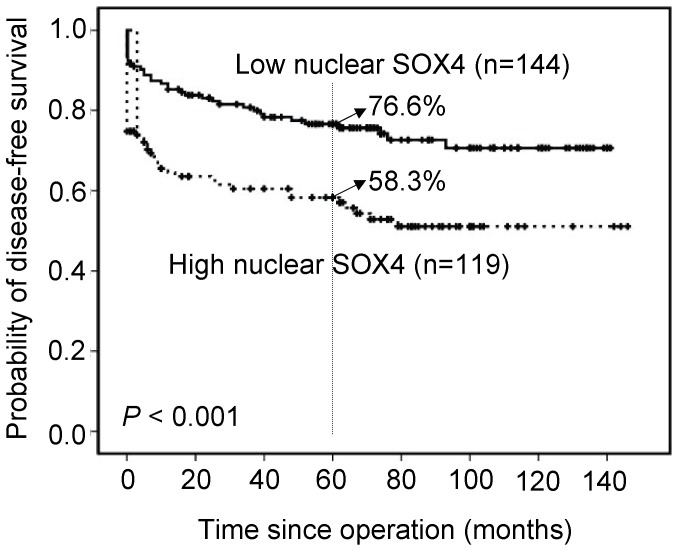
Overall survival analysis for 263 patients, stratified by nuclear SOX4 immunoreactivity (low nuclear SOX4: score = 0 or 1; high nuclear SOX4: score = 2 or 3). All statistical tests were two-tailed and the significance level was *P*<0.05.

**Figure 3:**
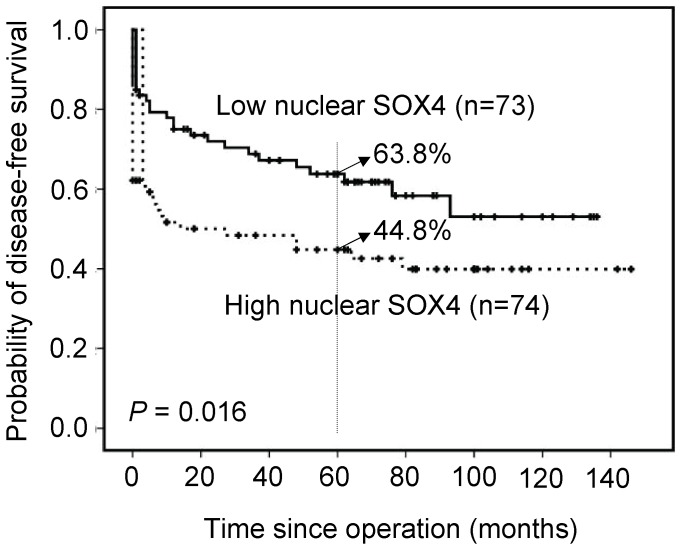
Disease-free survival analysis for 147 high-stage GC patients, stratified by nuclear SOX4 immunoreactivity (low nuclear SOX4: score = 0 or 1; high nuclear SOX4: score = 2 or 4). All statistical tests were two-tailed and the significance level was *P*<0.05.

Associations of clinical outcomes with nuclear SOX4 expression are shown in Figure 2. Overexpression of nuclear SOX4 was significantly associated with shorter disease-free survival (*P*<0.001). Patients with high expression levels of nuclear SOX4 achieved a 5-year disease-free survival rate of 58.3% compared with 76.6% for patients with low expression levels. Furthermore, high-stage GC (stage III and IV) was used to find out the effect of nuclear SOX4 overexpression on the prognosis. Overexpression of nuclear SOX4 was significantly associated with shorter disease-free survival (*P* = 0.016, Figure 3). Patients with high expression levels of nuclear SOX4 achieved a 5-year disease-free survival rate of 44.8% compared with 63.8% for patients with low expression levels.

The results of univariate analysis of the prognostic markers of colon cancer are shown in Table 4. Disease-free survival was significantly correlated with each of the following: depth of invasion (*P* = 0.029), nodal status (*P*<0.001), distant metastasis (*P*<0.001), stage (*P*<0.001), vascular invasion (*P*<0.001), perineural invasion (*P*<0.001), and overexpression of nuclear SOX4 (*P* = 0.001). However, the association between overexpression of nuclear SOX4 and survival was not significant after controlling for other well-known prognostic markers in multivariate analysis (*P* = 0.425, Table 5). In multivariate analysis, distant metastasis (HR = 0.052, 95% CI = 0.024 to 0.111, *P* = <0.001) was prognostically independent.

**Table 4:** Univariate analysis of prognostic markers in 263 patients with colon cancer.

Variable	HR (95% CI)*	*P* *
Depth of invasion	0.365 (0.148–0.901)	0.029
T1+ T2 (n = 33)		
T3+ T4 (n = 230)		
Nodal status	2.322 (1.481–3.642)	<0.001
N0 (n = 126)		
N1+ N2+ N3 (n = 137)		
Distant metastasis	21.284 (12.151–37.283)	<0.001
Absent (n = 224)		
Present (n = 39)		
Stage	3.654 (2.195–6.085)	<0.001
I+II (n = 116)		
III+IV (n = 147)		
Degree of differentiation	20.856 (0.031–14030.457)	0.361
Poor (n = 16)		
Well to moderate (n = 247)		
Vascular invasion	3.055 (1.893–4.931)	<0.001
Absent (n = 124)		
Present (n = 139)		
Perineural invasion	2.318 (1.487–3.614)	<0.001
Absent (n = 206)		
Present (n = 57)		
Nuclear SOX4	2.093 (1.366–3.206)	0.001
Low expression (n = 144)		
High expression (n = 119)		

* All statistical tests were two-tailed and the significance level was *P*<0.05. HR = hazard ratio; CI = confidence interval.

**Table 5:** Multivariate analysis of prognostic markers in 263 patients with colon cancer.

Variable	Adjusted HR (95% CI)* ^,^ #	*P* *
Depth of invasion	0.572 (0.225–1.456)	0.241
T1+ T2 (n = 33)		
T3+ T4 (n = 230)		
Nodal status	0.476 (0.204–1.111)	0.086
N0 (n = 126)		
N1+ N2+ N3 (n = 137)		
Distant metastasis	0.052 (0.024–0.111)	<0.001
Absent (n = 224)		
Present (n = 39)		
Stage	1.375 (0.485–3.895)	0.549
I+II (n = 116)		
III+IV (n = 147)		
Degree of differentiation	0.000 (0.000–253E250)	0.970
Poor (n = 16)		
Well to moderate (n = 247)		
Vascular invasion	0.673 (0.385–1.177)	0.165
Absent (n = 124)		
Present (n = 139)		
Perineural invasion	1.074 (0.646–1.786)	0.782
Absent (n = 206)		
Present (n = 57)		
Nuclear SOX4	0.829 (0.522–1.315)	0.425
Low expression (n = 144)		
High expression (n = 119)		

* All statistical tests were two-tailed and the significance level was *P*<0.05.

# Adjusted HR was obtained by depth of invasion, nodal status, distant metastasis, stage, degree of differentiation, vascular invasion, perineural invasion, and nuclear SOX4 expression.

## Discussion

Colon cancer remains a major public health problem worldwide [1]. In spite of the current surgical techniques and chemotherapy that have made significant improvements, the cure rate for advanced colon cancer remains low and the morbidity remains high [2]. Greater knowledge of the molecular mechanisms underlying the development of this deadly neoplasm is required if novel strategies to prevent and treat colon cancer are to be developed. In particular, identification of molecules that are altered during cancer initiation and progression can provide valuable tools as prognostic markers or therapeutic targets.

The expression of SOX4 in human cancers varies according to cancer type. The SOX4 level is elevated in numerous human cancers, including of the bladder, prostate, endometrium, and liver, whereas it is decreased in melanoma and gallbladder cancer [17], [18], [24]–[27]. In the present study, we assessed the expression levels of nuclear SOX4 in colon tissues obtained from 263 patients with colon cancer. Our results were consistent with those in bladder and prostate cancers, and showed that nuclear SOX4 expression was elevated in colon tumor tissues relative to non-tumor colon tissues. The immunoblotting results confirmed that nuclear SOX4 expression was higher in colon cancer cells than in normal colon cells.

Our finding also showed that overexpression of nuclear SOX4 in colon cancer tissues was closely correlated with tumor invasion and metastasis. The mechanism by which SOX4 exerts its invasive and metastatic activity remains unclear. In the first line of evidence, miRNAs (small noncoding RNAs with regulatory functions) were shown to be associated with tumor invasion and metastasis [28], [29]. Previous work by Tavazoie et al. showed that miR-335 suppresses metastasis through down-regulation of SOX4 [22]. This result suggested that SOX4 is linked to tumor aggressiveness.

The second line of evidence has been provided by studies on epithelial-mesenchymal transition (EMT), which is a key step during embryogenesis [30]. Accumulating evidence suggests that inappropriate utilization of EMT might be a component of the invasion of many tumors of epithelial tissues. Cell characteristics are strongly affected during EMT, resulting in alterations to cell-cell and cell-matrix interactions, cell motility, and invasiveness [31], [32]. Recent study of Zhang et al. showed that overexpression of SOX4 in human mammary epithelial cells led to the acquisition of mesenchymal traits, and enhanced cell migration and invasion. Furthermore, SOX4 positively regulated the expression of known EMT inducers and activated the TGF-β pathway to contribute to EMT. The expression of SOX4 was induced by TGF-β and was necessary for TGF-β-induced EMT. These findings show that SOX4 plays an important role in the progression of breast cancer, by orchestrating EMT [33]. These studies may account in part for the association of overexpression of nuclear SOX4 with tumor invasion and metastasis.

Precise prediction of the risk of recurrence would assist in minimizing the adverse effects of colon cancer and maximizing the therapeutic effect of treatment. Of the available prognostic markers for colon cancer, the AJCC stage is most important. However, the prognosis varies even among patients at the same disease stage; hence, alternative prognostic markers are sought. Few studies have investigated the prognostic value of SOX4 proteins. Aaboe et al. showed that a strong association existed between increased SOX4 expression and increased patient survival in cases of bladder cancer [17]. Similarly, Kim et al. showed that overexpression of SOX4 protein in patients with hepatocellular carcinoma was associated with improved patient outcomes [25]. In addition, Jafarnejad et al. showed that in melanoma patients, a strong association existed between reduced SOX4 expression and poor patient survival [26]. We found no published reports discussing the prognostic significance of SOX4 in human colon cancer. The results of this study showed that nuclear SOX4 overexpression was inversely correlated with patient survival; this finding contradicted the previously reported positive associations. Our study was the first to show that overexpression of nuclear SOX4 can predict poorer outcomes for patients with colon cancer. Overexpression of nuclear SOX4 appears to be a useful marker to predict outcomes in patients with colon cancer who have received surgical resection of the tumor. Thus, patients with colon cancer who display overexpression of nuclear SOX4 should be followed up carefully. Because our patient group was small, future studies should include a larger colon cancer patient group to elucidate the prognostic significance of nuclear SOX4 in this disease.

In summary, this study provided evidence for the clinical significance of overexpressed SOX4 in patients with colon cancer. Our findings indicate that targeting SOX4 might provide a new therapeutic modality for the treatment of colon cancer.
